# Study of Peripheral Microcirculation Assessed by Nailfold Video-Capillaroscopy and Association with Markers of Endothelial Dysfunction and Inflammation in Rheumatoid Arthritis

**DOI:** 10.31138/mjr.33.3.375

**Published:** 2022-09-30

**Authors:** Elena Angeloudi, Eleni Bekiari, Eleni Pagkopoulou, Panagiota Anyfanti, Michael Doumas, Alexandros Garyfallos, Theodoros Dimitroulas

**Affiliations:** 1 Second Department of Internal Medicine, Hippokration Hospital, Aristotle University of Thessaloniki, Greece,; 2 Fourth Department of Internal Medicine, Hippokration Hospital, Aristotle University of Thessaloniki, Greece,; 3 Second Propaedeutic Department of Internal Medicine, Hippokration Hospital, Aristotle University of Thessaloniki, Greece

**Keywords:** microcirculation, nailfold videocapillaroscopy, rheumatoid arthritis, endothelial dysfunction, atherosclerosis, inflammation markers

## Abstract

Rheumatoid Arthritis (RA) is a chronic systemic autoimmune disease that primarily affects synovial joints and is associated with increased cardiovascular (CV) mortality and morbidity. This association is only partially attributed to the presence of classic CV disease risk factors, and is strongly associated with characteristics of disease itself namely systemic chronic inflammation and autoimmune activation. Growing evidence suggests that microvascular endothelial dysfunction contributes to the initiation and progression of vascular disease. Nailfold capillaroscopy is a non-invasive method that evaluates the morphology and the structure of nailfold capillaries. Extension of this method is the Nailfold Videocapillaroscopy (NVC), which provides the possibility of combining functional and anatomical study of peripheral microcirculation. The present cross-sectional study aims to evaluate using NVC the peripheral microcirculation in adult patients with RA and investigate the associations between structural and functional indices of digital capillaries with markers of atherosclerosis.

## INTRODUCTION

Rheumatoid Arthritis (RA) is a chronic, systemic autoimmune disease characterized by persistent inflammatory synovitis that usually affects peripheral joints with symmetrical distribution.^1^ RA has been associated with reduced survival by 3–18 years compared to the general population^2^ with the leading cause of premature death being CV disease.^3^ In a meta-analysis involving 41,490 patients with RA, the risk of CV disease was 48% higher in patients with RA compared to the general population.^4^ These rates appear to be only partially correlated with the presence of classic cardiovascular risk factors, such as dyslipidaemia, hypertension, and smoking, thus indicating RA itself as an independent CV disease risk factor.^5^ The aetiology of high CV risk in RA is not fully understood.^6–8^ However, RA related chronic high grade systemic inflammation exerts detrimental effects on endothelium haemostasis such as reduction of nitric oxide production, dysregulation of vascular tone and activation of endothelial cells all of which culminate in atherosclerosis.^9^ In RA, vascular injury affects both micro- and microvasculature although most studies have focused mostly on macrovascular atherosclerotic coronary heart disease.^10–12^

The better understanding of the relationship between vascular wall damage and systemic inflammation, as well as the study of early atherosclerotic disease in RA have gained great research interest to the extent that RA is considered the most studied systemic inflammatory disease in this field. In addition, the presence of subclinical atherosclerosis in RA is common and until recently underestimated in clinical practice. The main cardiovascular risk assessment indices used primarily in patients with RA are Pulse Wave Velocity (PWV) and Carotid Intima-Media Thickness (cIMT).^13,14^ In view of microcirculation several studies and metanalyses have shown impairment of vascular function which in some cases may precede macro-vascular atherosclerotic disease.^15–18^ Nailfold capillaroscopy is a non-invasive, reproducible capillary imaging technique that examines the morphology and number of capillaries using a microscopic magnification system after placing a drop of cedar oil on the skin.^19^ NC is an established method for the diagnostic work out of patients with Raynaud’s and its implications are currently expanding in other clinical aspects of systemic sclerosis patients such as lung and cardiac involvement.^20^

An extension of the method of simple capillaroscopy is the video-capillaroscopy (NVC), which provides the possibility of structural and functional assessment of the microcirculation in the digital capillaries. The method enables the representation of the differences, in relation to rest, in the number of non-perfused capillaries after venous congestion or reactive hyperaemia after arterial occlusion (post-occlusive reactive hyperaemia). Thus, functional changes of the capillaries, in addition to the structural ones, are documented. For example, patients with arterial hypertension, who were the first group of non-rheumatic patients to have their microcirculation studied, showed reduced capillary density, both at rest and during venous congestion or after arterial occlusion.^21–26^

A few studies have investigated peripheral microcirculation in RA individuals. Rajaei et al.^27^ studied 430 patients with RA and recorded their capillary findings. The researchers observed an increased frequency of morphological capillary changes, in particular increased angiogenesis and severe capillary distortion (tortuosity). An earlier study^28^ also found that severe distortion and elongated capillaries are the main features of patients with RA, which concurs with previous observation,^29,30^ demonstrating low rates of scleroderma morphological lesions in patients with RA. Lin et al.^31^ also showed that the most prominent capillary finding in RA is increased capillary distortion and elongated capillaries. However, the association between NVC morphologic and functional abnormalities with indices of macrovascular disease in this population has not been extensively investigated. The current study aims to evaluate peripheral microcirculation assessed by NVC, in patients with RA and to explore the potential associations between structural and functional alterations of digital capillaries with markers of atherosclerosis such as cIMT and arterial stiffness.

## METHODS

### Study Design and Setting

This is a prospective cross-sectional observational study, which will be conducted in the Fourth Internal Medicine Department at Hippokration General Hospital of Thessaloniki in collaboration with the Hypertension Laboratory of the Second Propaedeutic Department of Internal Medicine, Hippokration General Hospital of Thessaloniki. The study received approval from the Aristotle University Ethics Committee. All participants will give informed consent before study entry.

#### Inclusion Criteria

This study enrols patients ≥ 18 years old with RA based on the criteria of the American College of Rheumatology and the European Society of Rheumatology (ACR / EULAR 2010) who are either already attending or attending for the first time the Rheumatology Division of the 4th Department of Internal Medicine, Hippokration General Hospital of Thessaloniki. Patients will have the full ability to understand and provide written signed consent to participate in the study after being informed.

#### Exclusion criteria

The doctoral dissertation protocol will exclude patients with known, confirmed by biopsy, active malignancy, patients with myocardial infarction or unstable angina episode in the last 3 months and/or congestive heart failure category IV, according to the criteria of New York Heart Association as well as patients who refuse to participate in the study and sign the consent form. Also, patients with diabetes mellitus, chronic kidney disease stage 4 or 5 with eGFR <30ml/min/1.73m2 for at least three months will be excluded as well as patients with a change in anti-hypertensive treatment in the last month before the start of the study. Finally, patients with previous vascular carotid surgery due to anatomical difficulty in assessing the cIMT index will be excluded.

#### Study Overview

Complete medical history, including medication record and smoking habits will be obtained from all participants. Patients will undergo clinical examination, office blood pressure measurement and blood sample collection for routine haematology, biochemistry including lipid profile and blood glucose and inflammatory markers. Centrifuged samples will be stored in a deep freezer (−70°C) to determine Asymmetric Dimethylarginine (ADMA) levels after collection of all samples.

Following the blood sampling, the patients will undergo tonometric measurement of arterial stiffness and ultrasound determination of cIMT using SphygmoCor and Vivid 7 of General Electric devices, respectively. Finally, the patient will be transferred to the Rheumatology Division of the Fourth Department of Internal Medicine, in order to have NVC with the use of the Optilia Digital Capillaroscope device. After the end of the evaluation, the patient will receive from the rheumatologist the medical instructions that correspond to the specific scheduled visit and the monitoring within the protocol will be ceased. All vascular assessments will be performed at the same time period for each patient within 24 hours before or after blood sample collection.

### Study Procedures

#### Measurements with the SphygmoCor device

The radial artery applanation tonometry method is used to record the pulse waveform in the carotid artery, using the SphygmoCor device, AtCor Medical Pty Ltd, Sydney, NSW, Australia. This method will calculate the augmentation index (AIx) based on the augmentation pressure (systolic pressure minus the inflection pressure) divided by the pulse pressure (systolic minus diastolic pressure), expressed as a percentage.^32^ As the AIx is dependent on the transit time of the reflected wave and the time of arrival of the reflected wave during the pressure pulse, it is also sensitive to heart rate. The SphygmoCor software reports the AIx at the patient’s current heart rate, as well as giving an AIx corrected to a heart rate of 75 bpm using a regression to the population heart rate dependency of the Aix.^33^ PWV will then be determined in each patient as a marker of arterial stiffness. The carotid and femoral pulse is acquired by applanation tonometry sequentially, allowing a single operator to acquire the measurement. The transit time from the R-wave of the simultaneously acquired electrocardiogram to the foot of the carotid and femoral pulse is measured.^34^ The difference between these 2 transit times is divided by distances measured from the body surface to estimate the arterial path length in order to calculate the carotid-femoral PWV. The length of the vascular pathway is calculated by measuring linear distances between the palpation point of the carotid artery with the supernatant incision and the supernatural incision with the palpation point of the femoral artery.^35^

#### cIMT Measurement

Ultrasound determination of cIMT of the two common carotid arteries will be performed using Vivid 7 of General Electrics based on the consensus of Mannheim.^36^ The cIMT measurement will be performed in a region free of plaque where there is a standard double-line pattern, and the measurements are more accurate and reproducible. The sections of the arterial walls will be evaluated in a longitudinal view, strictly perpendicular to the ultrasound beam, with both walls being visible to achieve diameter measurements.^37^ The optimal diameter will be obtained during dilation by looking for the minimum diameter during the cardiac cycle. The cIMT will be measured on the proximal vessel wall, as the cIMT values of the distal wall are less reliable. At least 10mm in length, an arterial section will take a high-resolution image to perform a series of reproducible measurements.^38^

#### Nailfold Videocapillaroscopy

NVC is an established non-invasive method to assess the capillary vasculature of the digital arteries, which provides information on the structural and functional abnormalities of the capillaries. It is performed at room temperature (22–23°C) with the patient seated and resting for 15 minutes NVC will be performed at the Fourth Internal Medicine Department at Hippokrateion Hospital using an Optilia Digital Capillaroscope. Pictures and videos of 15 seconds duration will be captured using the 200x magnification video camera from the second to the fifth finger of each hand. The images will be analysed with Optipix capillaroscopy software 1.7.x on a computer. The NVC parameters to be measured are: capillary density, capillary width and length (μm), the presence of micro-bleeding, oedema, thrombi, and the presence of any abnormalities in the morphology or disorder of capillary architecture. These parameters will be determined on each nail (except thumbs) and the average of all measurements for each patient will be calculated at the end.^39^ In addition to the quantitative and qualitative parameters, a functional evaluation of the changes in capillary density will be performed using digital video in three phases, as follows: the capillaries will be imaged on the skin of the distal phalanges of the dorsal region of the third and fourth fingers of the two upper limbs. The imaging will be about 4.5mm near the terminal row of capillaries in the middle of nailfold. The researcher will select a skin area of 1 mm^2^. Capillary density will be measured in three conditions: a) baseline capillary density, which is defined as the number of continuously filled red blood cells per 1 mm^2^ of skin and measured for 15 seconds; b) capillary density during hyperaemia after arterial occlusion, which is evaluated after 4 minutes of arterial occlusion [use a microscopic finger cuff applied to the base of the finger under study and ‘filled’ at hypersystolic pressure (260mmHg) for 4 minutes]. Immediately after the release of the cuff, all (continuously and intermittently) paid capillaries are counted for 15 seconds and c) venous congestion density using a cuff at 60mmHg for two minutes and then all (continuously and intermittently) capillaries will be measured for 15 seconds.^40–43^

### Outcomes

#### Primary outcomes

- Investigation of the correlation between capillary density at baseline, after 4-min arterial occlusion and after 2-min venous occlusion, with atherosclerosis indices (cIMT, PWV, AIX)

#### Secondary Outcomes

- Investigation of the correlation between microangiopathy findings, as assessed by video-capillaroscopy, and biochemical markers of endothelial function, such as ADMA, CRP, and uric acid
- Investigation of the correlation between microangiopathy findings, as assessed by video-capillaroscopy, and overall cardiovascular risk

#### Sample Size

Published studies^44^ have indicated a statistically significant correlation between basal capillary density and markers microvascular disease in patients with systemic sclerosis (Pearson’s correlation coefficient R = 0.35, p-value = 0.044). In the international literature^45,46^ it has a significant correlation of capillary density with factors such as gender, age, smoking, hypertension, and diabetes, while the possible effect of taking organic factors as the main medication. According to the above, one sample of 105 people has 80% statistical power to detect an expected effect size equal to 0.15 in a multiple regression model with 7 independent variables assuming type I error, α = 0.05.^47–50^ In addition, to ensure the completeness of all statistical checks our sample will includes at least ten people in each one (one in ten rule)^51^ from the above risk factors (gender, age group, smokers, people with hypertension, people with diabetes).

## ANTICIPATED BENEFITS

The research hypothesis that in patients with RA a correlation is observed between the parameters of NVC with markers of endothelial dysfunction and inflammation, may contribute to the introduction of a new non-invasive reliable method for assessing the severity and progression of the peripheral microvascular disease in patients with RA. It will also help to understand the pathophysiology of vascular damage at the microvascular level and may be able to contribute to better assessment of CV risk in patients with RA. This may extend the use of NVC, as a reliable, reproducible and at the same time simple and non-invasive, complementary method for the assessment stratification of CV risk in this population.
